# Frontiers of Robotic Gastroscopy: A Comprehensive Review of Robotic Gastroscopes and Technologies

**DOI:** 10.3390/cancers12102775

**Published:** 2020-09-28

**Authors:** Wojciech Marlicz, Xuyang Ren, Alexander Robertson, Karolina Skonieczna-Żydecka, Igor Łoniewski, Paolo Dario, Shuxin Wang, John N Plevris, Anastasios Koulaouzidis, Gastone Ciuti

**Affiliations:** 1 Department of Gastroenterology, Pomeranian Medical University in Szczecin, 71-252 Szczecin, Poland; marlicz@hotmail.com; 2 The Centre for Digestive Diseases Endoklinika, 70-535 Szczecin, Poland; 3 The BioRobotics Institute, Scuola Superiore Sant’Anna, 56025 Pisa, Italy; paolo.dario@santannapisa.it; 4 Department of Excellence in Robotics & AI, Scuola Superiore Sant’Anna, 56127 Pisa, Italy; 5 Key Laboratory of Mechanism Theory and Equipment Design of Ministry of Education, Tianjin University, Tianjin 300350, China; shuxinw@tju.edu.cn; 6 Department of Gastroenterology, Western General Hospital, Edinburgh, Scotland EH4 2XU, UK; alexander.robertson1@nhs.net; 7 Department of Human Nutrition and Metabolomics, Pomeranian Medical University in Szczecin, 71-460 Szczecin, Poland; karzyd@pum.edu.pl (K.S.-Ż.); igor.loniewski@sanprobi.pl (I.Ł.); 8 Endoscopy Unit, The Royal Infirmary of Edinburgh, Edinburgh, Scotland EH16 4SA, UK; j.plevris@ed.ac.uk (J.N.P.); akoulaouzidis@hotmail.com (A.K.); 9 Medical School, the University of Edinburgh, Edinburgh, Scotland EH8 9AG, UK

**Keywords:** gastroscopy, gastric cancer, robotic gastroscopy, machine learning, artificial intelligence

## Abstract

**Simple Summary:**

With the rapid advancements of medical technologies and patients’ higher expectations for precision diagnostic and surgical outcomes, gastroscopy has been increasingly adopted for the detection and treatment of pathologies in the upper digestive tract. Correspondingly, robotic gastroscopes with advanced functionalities, e.g., disposable, dextrous and not invasive solutions, have been developed in the last years. This article extensively reviews these novel devices and describes their functionalities and performance. In addition, the implementation of artificial intelligence technology into robotic gastroscopes, combined with remote telehealth endoscopy services, are discussed. The aim of this paper is to provide a clear and comprehensive view of contemporary robotic gastroscopes and ancillary technologies to support medical practitioners in their future clinical practice but also to inspire and drive new engineering developments.

**Abstract:**

Upper gastrointestinal (UGI) tract pathology is common worldwide. With recent advancements in robotics, innovative diagnostic and treatment devices have been developed and several translational attempts made. This review paper aims to provide a highly pictorial critical review of robotic gastroscopes, so that clinicians and researchers can obtain a swift and comprehensive overview of key technologies and challenges. Therefore, the paper presents robotic gastroscopes, either commercial or at a progressed technology readiness level. Among them, we show tethered and wireless gastroscopes, as well as devices aimed for UGI surgery. The technological features of these instruments, as well as their clinical adoption and performance, are described and compared. Although the existing endoscopic devices have thus far provided substantial improvements in the effectiveness of diagnosis and treatment, there are certain aspects that represent unwavering predicaments of the current gastroenterology practice. A detailed list includes difficulties and risks, such as transmission of communicable diseases (e.g., COVID-19) due to the doctor–patient proximity, unchanged learning curves, variable detection rates, procedure-related adverse events, endoscopists’ and nurses’ burnouts, limited human and/or material resources, and patients’ preferences to choose non-invasive options that further interfere with the successful implementation and adoption of routine screening. The combination of robotics and artificial intelligence, as well as remote telehealth endoscopy services, are also discussed, as viable solutions to improve existing platforms for diagnosis and treatment are emerging.

## 1. Introduction

Upper gastrointestinal (UGI) tract disorders are global and common diseases [1]. Since 1990, the age-standardised rates of stomach cancer have declined worldwide; however, stomach cancer holds onto a poor prognosis when compared to many other malignancies and ranks fifth and third in incidence and mortality among cancers, worldwide [2,3]. Furthermore, oesophageal cancer not only has an unfavourable prognosis but also remains the sixth leading cause of death worldwide [4]. Recent advancements in both robotics and Artificial Intelligence (AI) allowed forward strides in the domains of diagnostic and treatment [5,6,7]. In countries where they are employed, UGI cancer screening programs have significantly improved diagnostic rates and mortality indices [8]. Nevertheless, the detection rate of early UGI tract cancer, with conventional esophagogastroduodenoscopy, remains low [9]. Although new online learning platforms have been introduced to help gastroenterologists to master their diagnostic skills in endoscopic detection of early UGI tract disease and to allow for more favourable outcomes [10], the roll-out of mass-screening is difficult and often hampered by a relatively low compliance rate [11]. Furthermore, meta-analyses demonstrated that around 10% of UGI cancers are missed during endoscopy exams performed within 3 years before diagnosis [12,13,14].

The diagnostic and therapeutic capabilities of UGI endoscopy strongly correlate with the technical and decision-making skills of the operator [15], as well as other variables such as: (i) risk of communicable disease transmission, i.e., viral infections, such as the severe acute respiratory syndrome COVID-19, which is very infectious through aerosol; (ii) unaltered learning curves to achieve proficiency in endoscopic techniques; (iii) increasing numbers of physicians/nurses with burnout or endoscopy-related injuries [16]; (iv) limited human and/or material resources; and (v) patients’ perspectives on conventional endoscopy of the UGI tract that further interfere with the successful implementation and adoption of routine screening [17]. UGI robotic endoscopes, assisted by AI, can augment both the diagnostic and therapeutic capability, as well as the precision of endoscopic interventions. For example, such robotic platforms can calculate the force that should be applied to the GI tract wall, as well as perform movements in three-dimensional mode when using operative tools, hence decreasing the probability of adverse events [18].

Undoubtedly, the development of UGI endoscopy robotic platforms finds its natural application in the field of endoscopic submucosal dissection (ESD). ESD requires a high level of endoscopic skill and control; consequently, its adoption in the field is slow. Although ESD has been initially linked with relatively high incidence of post-intervention complications [19], the overall current outcomes are better than the ones of radical surgery [20,21]. As ESD enables minimally invasive, successful and endoscopically complete (R0) removal of early gastric cancers and other malignant and non-malignant submucosal lesions located in the oesophagus or the stomach, it should be considered as the treatment of choice for certain early UGI tract disease [22,23]. Robotics can enhance en bloc lesion removal and histological completeness of the endoscopic resection [19]; therefore, ESD could accrue a second wave of wider adoption with a dedicated robotic support [18,24]. Furthermore, Natural Orifice Transluminal Endoscopic Surgery (NOTES), as well as bariatric surgery, could benefit from robotic platforms, due to their functional versatility [25,26]. Over recent years, many robotic endoscopes for the UGI tract have been designed and developed but only a few have reached the market [27]. Admittedly, robotic endoscopes are potentially costly and a niche market in healthcare provision; however, acquisition costs can be off-set by a higher precision in care delivery, and easier adoption of more precise endoscopic interventions [28].

In this review, authors will first present a short overview on current endoscopy practices for early detection of UGI cancerous and non-cancerous lesions followed by the recent advances and challenges in the field of robotic gastroscopy. We will focus on the early detection and follow up of these lesions in the practice of luminal GI endoscopy, and subsequently compare the capabilities of robotic technologies with contemporary basic quality requirements for standard endoscopy. We will also briefly discuss the limitations of current GI endoscopy practices at a time of pandemic and call for innovative solutions, including robotic (capsule) UGI screening platforms combined with telemedicine and remote healthcare services. This is clustered into five principal classes: (i) commercially available gastroscopes (Section 2); (ii) research-oriented robotic gastroscopes (Section 3); (iii) novel flexible robots for upper-GI tract surgery (Section 4); (iv) artificial intelligence in gastroscopy (Section 5), and (v) finally we will briefly discuss robotic UGI screening combined with remote healthcare services (Section 6).

## 2. Commercially Available Gastroscopes: Conventional Instruments and Robotic Platforms

### 2.1. Diagnosis of UGI Malignant and Non-Malignant Lesions with Conventional Gastroscopes

**Figure 1:**
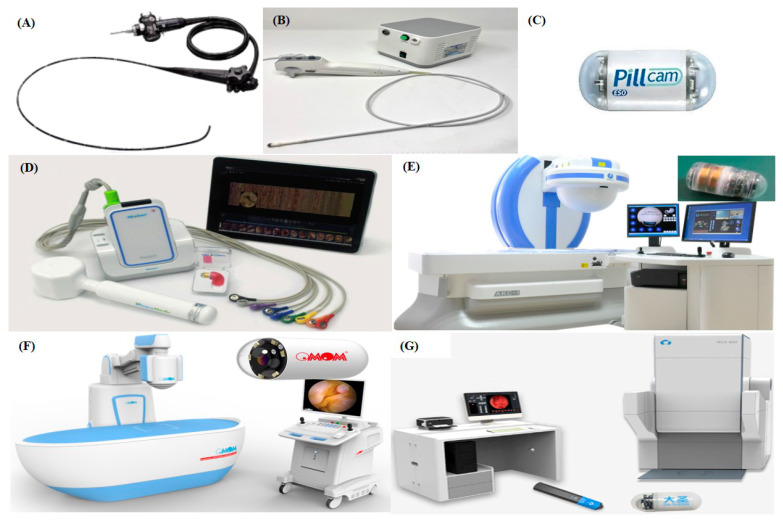
Commercially available gastroscopes: (**A**) Olympus gastroscope (courtesy of Olympus Corp.) [31]; (**B**) EG Scan gastroscope (IntroMedic, Seoul, Korea; courtesy of IntroMedic Ltd.); (**C**) PillCam^TM^ capsule endoscope [35,36,37]; (**D**) MiroCam-Navi system by Intromedic Ltd. (Seoul, Korea) [38]; (**E**) Navicam by Ankon Technologies Co., Ltd. (Wuhan, Shanghai, China; courtesy of ANKON Co., Ltd.) [39]; (**F**) OMOM Capsule Endoscopy platform by Jinshan Science & Technology (Group) Co., Ltd. (Chongqing, China;courtesy of Jinshan Science & Technology (Group) Co., Ltd.) [40]; (**G**) Standing-type magnetically controlled capsule endoscopy system by JIFU Medical Technologies Co., Ltd. (Shenzhen, China) [41].

The progression to gastric cancer is preceded by chronic gastritis, gastric atrophy, gastric intestinal metaplasia and dysplasia. A crucial factor contributing to early detection of cancer and improved survival is non-invasively identifying those at risk [29]. The clinical data on the use of non-invasive biomarkers to identify patients with chronic atrophic gastritis is scarce with no data supporting a screening of the population. Therefore, a high-quality endoscope with complete mucosal visualization is an integral part in improving the early detection of UGI lesions. Image-enhanced endoscopy combined with biopsy sampling for histopathology is currently the best approach to detect and accurately risk-stratify patients at risk. Biopsies following the Sydney protocol from the antrum, incisura, lesser and greater curvature allow both diagnostic confirmation and risk stratification in regard to cancer progression [29]. Moreover, all UGI lesions should be endoscopically identified with measurable quality indicators, such as time recording, photo documentation, and the description of lesions utilizing standard terminology [30]. To this effect, several conventional gastroscopes with a wide range of technological features are available. Most modern UGI endoscopes from well-established manufacturers come with small diameter (<10 mm) and large bending angle (up to 210 degrees) (Figure 1A) [31], allowing high-powered optical functions, e.g., powerful white light source, high-definition, near focus, auto-fluorescence imaging, narrow band imaging, spectral imaging colour enhancement-based modalities or even AI capabilities. A new exemplary is the disposable gastroscope free of disinfection procedure (Figure 1B).

Of importance is that the position statements on quality standards in UGI endoscopy issued by the British Society of Gastroenterology, Association of Upper Gastrointestinal Surgeons of Great Britain and Ireland, as well as other gastrointestinal societies [32,33,34], aimed to reduce the variation in practice and standards between individual endoscopists and entire units by establishing a set of auditable key performance indicators [14]. In particular, these recommendations aim to optimize the diagnosis of early neoplasia and premalignant conditions, in order to favourably alter the natural history of UGI malignancies. Future robotic gastroscopes equipped with AI-based software modules could aim at a significant reduction in the variation of lesion assessment and reduce the bias associated with the human factor.

### 2.2. Gastric Capsule Endoscopes

#### 2.2.1. UGI Motility-Actuated Capsule Gastroscopes

Alongside conventional and innovative flexible gastroscopes, several manufactures have developed passive wireless capsule endoscopes (WCEs). The first commercially available WCE for UGI screening, the PillCam^TM^ ESO capsule, was introduced by Given Imaging Ltd. (Yokneam Illit, Israel) and received the U.S. Food and Drug Administration (FDA) clearance in 2004 (Figure 1C) [35]. The ingestible, disposable PillCam^TM^ ESO has a small dimension (11 × 26 mm), and its main feature was the presence of a second optical dome and an increased image acquisition frame rate (14 frame per second - FPS), as compared to conventional small-bowel WCEs (2 FPS). However, this is at the expense of a shorter battery life offering, therefore, limited inspection of the stomach and of the duodenum. The upgraded version, ESO2, has the same dimensions bur higher image acquisition frame rate (18 FPS); the operating time was increased to 30 min [36]. Over the years, the use of the ESO capsules has waxed and waned due to inferior image quality than conventional endoscopes, the lack of steerability and movement control, lack of air insufflation/aspiration and complex/variable patient movement protocols. Nevertheless, despite its inherent limitations, the principal contribution of this passive capsule device was the proof of concept that wireless examination of the UGI tract could be feasible and promising.

Its successor, the improved version embodied in PillCam^TM^ UGI, has been part of Medtronic Plc.’s (Dublin, Ireland) armamentarium since 2015. This novel device has similar dimensions (11.6 × 32.8 mm) with a wide field of view (172 degrees for each of the 2 cameras), an adjustable frame acquisition rate (4-35 FPS) and an operating time of 10 h [37]. The PillCam^TM^ UGI capsule is, therefore, an attractive alternative to conventional gastroscopy as it can provide a discomfort- and sedation-free procedure. However, it still offers a limited view within the stomach due to a non-active control of the device, as in the previous versions of wireless capsule gastroscopes.

#### 2.2.2. Externally Actuated Capsule Gastroscopes

Using an external magnetic field to steer and drive a WCE was one of the solutions proposed to improve the diagnostic performance. A complete gastric mucosal examination becomes possible by employing a navigation system that guides the active WCE for a more accurate and reliable control of the capsule gastroscope in the gastric cavity. The MiroCam-Navi system, developed by Intromedic Ltd. (Seoul, Korea), was designed by modifying the standard MiroCam^®^ SB capsule including a permanent magnet, hence allowing a controlled exploration of the gastric cavity (Figure 1D) [38]. Steering is controlled by a hand-held, hammer-like, permanent magnet and the images from the WCE are transmitted to a real-time viewing device via wireless communication from an external belt. The effectiveness of this device had been validated [42]. Hale et al. demonstrated that the MiroCam-Navi system had comparable detection rates with respect to conventional gastroscopes (89% versus 88%) in identifying beads sewn onto the mucosal surface of ex-vivo porcine stomachs [43].

Ankon Technologies Co., Ltd. (Wuhan, Shanghai, China) developed a magnetically controlled capsule endoscope (Navicam, MCE) aimed at exploring the stomach for gastric diseases screening (Figure 1E). It consists of a WCE, a magnetic-guidance robot, a data recorder and a computer workstation for real-time viewing and capsule navigation control. The safety and feasibility of the system has been validated by the stomach inspections of 34 human volunteers with the examination well accepted by these subjects without adverse events. A complete stomach examination takes on average 43.8 ± 10.0 min (range: 27–60 min) [39]. The examination of the stomach in 350 patients (mean age: 46.6 years), was performed safely and successfully, showing comparable accuracy with respect to conventional gastroscope. The MCE did not miss any significant lesions (including tumours and large ulcers) [44]. This system was clinically approved by the China Food and Drug Administration (CFDA mark—registration number: 20173223192) in 2013 and then obtained the European Union CE certification (CE mark) [45]. Using an updated version of this system, Ching et al. reported 100% views of the gastric cardia, fundus, greater and lesser curvature, anterior and posterior wall, antrum and pylorus, and furthermore, the capsule gastroscope could continue through peristalsis to image small intestine following the examination of stomach [46].

Another system, developed by Jinshan Science & Technology (Group) Co., Ltd. (Chongqing, China), is a magnetic robotic platform for gastroscopy, i.e., the OMOM Capsule Endoscopy platform. The system includes a disk-shaped permanent magnet controller held above an operating bed and a diagnostic capsule containing permanent magnets (Figure 1F) [40]. The OMOM capsule is light (i.e., 6 g), compact (i.e., 13 × 27.9 mm) and integrates a high-qualified image sensor (512 × 512 resolution, adaptive 2 to 6 FPS and field of view of 172 degrees). The high-performance Light Emitting Diode (LED) system ensures a wide and balanced light distribution and optimal illumination in a tubular environment. In addition, the battery life is more than 12 h.

Furthermore, a standing type, magnetically controlled WCE system has been developed by JIFU Medical Technologies Co., Ltd. (Shenzhen, China). It comprised a WCE, a guidance magnetic robot, and an imaging computer. It was also able to inspect the gastric cavity including the entire cardia and fundus and screen gastric illnesses without any anaesthesia (Figure 1G). A prospective multicentre, blinded study, that compared standing type magnetically controlled WCE with conventional gastroscope in patients, was carried out. Overall, the compliance rate was 94.41%, and 64 pathological outcomes were identified (50 outcomes were detected by both, 7 by standing type magnetically controlled WCE and 7 by conventional gastroscope). Standing type magnetically controlled WCE provides equivalent agreement with the gastroscope [41].

## 3. Research-Oriented Robotic Gastroscopes: Innovative Smart Devices for Upper-GI Tract

### 3.1. Tethered Capsule-Like Gastroscopes

Gastroscopy remains a challenging procedure for patients and clinicians alike. Conventional endoscopy innovations, e.g., as ultrathin gastroscopes and trans-nasal endoscopy, allow an easier and distress-free approach. Therefore, further innovation and improvement of gastroscopes is an active area of research. Aside from the commercially available gastroscope, researchers seek to diagnose the gastric mucosa and propose innovative devices with alternative propulsion and bending mechanisms.

**Figure 2:**
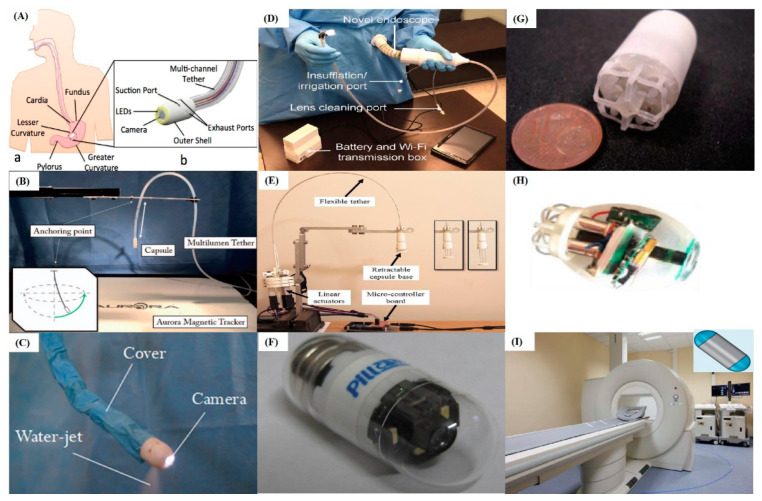
Research (non-commercialized) robotic gastroscopes: HydroJet-like gastroscopes developed by (**A**) Caprara et al. [47], (**B**) Campisano et al. [48], and (**C**) Yin et al. [49]; (**D**) Pneumatic gastroscope developed by Garbin et al. [50]; (**E**) Cable driven gastroscope developed by Ye et al. [51]; (**F**) Gastroscope modified by Swain et al. [52]; Self-driven gastroscopes developed by (**G**) Tortora et al. [53], and (**H**) De Falco et al. [54]; (**I**) Magnetic steering devices developed cooperatively by Olympus Corp. (Tokyo, Japan) and Siemens Healthcare GmbH (Erlangen, Germany) [55].

In 2015, Caprara et al. introduced a flexible gastroscope driven by internal waterjet actuation (HydroJet capsule gastroscope—Figure 2A) [47]. The system consisted of a swallowable capsule endoscope connected to an external water distribution system through a multi-channel soft tether. The camera, placed at the tip of the gastroscope, is oriented by activating the different waterjet channels with a variable pressure ejected from the capsule’s head. The capsule was able to visualize the cavity of the stomach by combining waterjet actuation with adjustments of the length of the tether. The system, validate in ex-vivo conditions, took less than 8 min to visualize the essential points of a typical gastric screening procedure. Campisano et al. in 2017, as part of the same research team, proposed an updated version of the HydroJet capsule gastroscope able to provide gastric cavity inspection (Figure 2B) [48]. The diameter of the capsule is smaller than the previous one because of the reduction of the number of actuating jets from 6 to 3. Because of this modification, the efficiency of the jet actuators was greatly improved, requiring less water. The water distribution system and the soft connected tether were also redesigned and manufactured to reduce the friction between the tether and the human tissue, allowing a greater degree of control. The trial was carried out in an anatomically realistic human stomach phantom to validate the feasibility of retroflexion for this capsule within a confined space. Results indicated that the presented HydroJet gastroscope was able to fulfil the medical requirements of inspecting the stomach adequately, although the time taken to perform the procedure was longer than the conventional UGI endoscopy: the time differences are 162 s for novices and 624 s for experts.

Combining thermo-sensitive shape memory material, a braided structure, and a water ejecting actuation, Yin et al. designed in 2018 a prototype of a robotic gastroscope, which is able to bend and fix the temporary shape (Figure 2C) [49]. The system can achieve a large workspace (length of 160 mm and width of 150 mm) by controlling the direction and pressure of the waterjet and a remarkable structure stiffness variability (stiffness ratio between rigid state and soft state is 16) by controlling the temperature of the water. The structure could be fixed as the thermosensitive material switches from soft state to rigid state. Experimental results have shown that it required 15 s to decrease from 37 °C to 5 °C to be rigid, and approximately 12 s to soften the prototype. In the phantom experiment, this device took longer than the endoscope to complete a trial: the average time for completion of a single trial after training was 242 ± 13 s by a gastroenterologist.

Differently from the Hydrojet solutions developed at the Vanderbilt University (Nashville, TN, USA) [47,48], that were based on the coupling between the mechanical joystick and pneumatic drive, Garbin et al. developed in 2019, as part of the same research team, an intuitive handling and portable device for the visual inspection of the gastric cavity (Figure 2D) [50]. The proposed device adopted a multi-backbone continuum structure as the user interface and parallel bellows’ actuators to manipulate the tip. The experiments in the human-like stomach phantom indicated that the device was able to efficiently find the LED markings of key anatomic landmarks. All landmarks were successfully identified with both endoscopes for all trials (*n*  =  900). Clinicians performed the procedure in a shorter time with the conventional endoscope when compared to this device (24.48 s versus 37.13 s), but this difference is within the acceptable range for clinical operations. There was no significant time difference between platforms for novices, suggesting that there was no significant difference in time between learning to master the novel device as compared to the conventional gastroscopes.

Finally, in 2017 Ye et al. presented a cable steered gastroscope, which consisted of an actuation section containing three motors, a thin and flexible tether with three super-elastic wires, and a retractable capsule base for installation of cameras (Figure 2E) [51]. With the lead screws, the rotation of the motors was transferred into the linear motion of the actuator rods, allowing bending in all directions to be achieved. This gastroscope was able to be extended to a distance of 40 mm and bend 360 degrees, radially, and up to 110 degrees, axially. In addition, the proposed gastroscope could be reconfigured to reach larger workspaces.

### 3.2. Wireless Capsule Gastroscopes

Without the constraints of cables, capsule gastroscopes were able to move and make diagnoses less invasive than tethered gastroscopes, so patients will be more comfortable during the entire examination.

In 2009, Tortora et al. presented a swallowable active capsule gastroscope composed of a supporting shell containing a wireless microcontroller, a battery and four brushed motors to activate rear independent propellers (Figure 2G) [53]. The diameter and the length of the device are 15 mm and 30 mm, respectively. The system was able to navigate in all directions in a water-filled area, achieving reliable locomotion and steering in the stomach. The capsule can guarantee diagnostic speed between 0 to 5–7 cm/s and is able to be actively controlled for more than 30 min at a selected diagnostic speed (i.e., 1.5 cm/s) with a mean current consumption below 40 mA. This system offered the potential for clinicians to inspect the gastric cavity in real-time. Through the human machine interface, clinicians will be able to control the capsule towards areas of interest using a triaxial joystick. The proposed system has been tested in in-vitro, ex-vivo and animal experiments. An advanced version of this capsule, with an embedded camera module, was developed by De Falco et al., as part of the same team, in 2014 (Figure 2H) [54]. The capsule gastroscope is 32 mm in length and 22 mm in diameter (still not suitable for being ingested), and it has been equipped with a miniaturized wireless vision system that acquires images with a frame rate up to 30 FPS. An explanted porcine stomach was used to evaluate the performance of the capsule in a compliant surrounding environment. The image transmission frame rate was 23–26 FPS in the ex-vivo environment, slightly lower than in in-vitro conditions due to the different tissue-interface. However, it was sufficient to allow real-time control of the active capsule and was higher than any other wireless vision system currently available. This capsule gastroscope has an operative lifetime at full charge of at least 13 min (i.e., with all components turned on and the transmission rate set at 30 FPS, that means a consumption of about 210 mA), close to the diagnostic operating time of the traditional gastroscopic examination.

Before the commercial magnetically driven wireless gastroscopes were applied, in order to manipulate the wireless gastroscope more feasibly and precisely in the gastric cavity, in 2010, the capsule pioneer Prof. Paul Swain and his colleagues modified a PillCam^TM^ WCE (Given Imaging Ltd., Yokneam Illit, Israel) to include neodymium-iron-boron magnets (Figure 2F) [52]. A handheld external magnet was used to manipulate this capsule gastroscope in the gastric cavity. The experiment indicated that the capsule gastroscope could be held and rotated at any positions in the stomach and moved back from pylorus to the cardio-oesophageal junction easily after the diagnosis. The safety and feasibility of the modified capsule gastroscope were validated in 2011. The magnetic manoeuvrable capsule was always attracted by the external magnetic paddle and responded to its movements; it remained in the stomach for 39 ± 24 min. In 7 subjects, both the cardia and the pylorus were inspected and 75% or more of the gastric mucosa was visualized. No adverse events were reported [56].

In 2010, a novel magnetic steering device for gastroscopy was developed cooperatively by Olympus Corp. (Tokyo, Japan) and Siemens Healthcare GmbH (Erlangen, Germany). The system included an Olympus Inc. WCE and a Siemens magnetic guidance equipment for interactively moving the capsule in the gastric cavity (Figure 2I) [55]. The capsule is controlled with a very low-intensity magnetic field (3–10 mT, 150 to 500 times smaller than that used for MRI) by the physicians using two joysticks and can be moved in the stomach with five independent degrees of freedom, i.e., translations, tilting and rotation. The in-vivo experiment in 53 subjects (29 volunteers and 24 patients) indicated that a sufficiently accurate gastric examination was feasible with this system: the technical success rate was 98%, and in the successful cases, examiners assessed that the antrum, body, fundus, and cardia were fully visualized in 98%, 96%, 73% and 75%, respectively. Mean duration of examinations was 30 min (range 8–50 min). The first blinded, comparative clinical trial in humans, which assesses the performance of magnetically guided capsule gastroscope versus a conventional high-definition gastroscope, was undertaken in 2011 in order to showcase the potentials of the wireless capsule gastroscope [57]. Visualization of the gastric pylorus, antrum, body, fundus, and cardia was evaluated as complete in 88.5%, 86.9%, 93.4%, 85.2%, and 88.5% of patients, respectively. Of the gastric lesions, 58.3% were detected by both gastroscopy and magnetically guided capsule endoscope at immediate assessment and review of recorded data. Capsule examination missed 14 findings and conventional gastroscopy missed 31 findings seen with magnetically guided capsule endoscope. Overall diagnostic yield was similar for both the modalities.

Finally, in 2012, Kósa et al. also developed an MRI-driven swimming capsule robot which had the potential application of inspecting the gastric cavity [58]. Two propelling methods, based on the MRI magnet, were tested and it was demonstrated that the high magnetic field of the MRI allows propulsion speed on the order of several millimetres per second.

## 4. Novel Flexible Robots for UGI Surgery

The increasing sophistication of robotic platforms, from experimental to commercially available systems, has fed the ongoing evolution of what is surgically possible. A drive towards minimally invasive procedures led to a desire for NOTES, “scar-less” surgery. Robotic NOTES is a natural progression of this. NOTES grants transluminal access to the peritoneal cavity with the aim of a “scar-less” intervention [59,60]. This is a fusion of surgical and endoscopic techniques and to achieve this requires a fine degree of accuracy and precision. Apart from NOTES, several articulated robots with surgical functionalities for UGI applications have been developed in the last year and are summarized in this section, after introducing the conventional gastric surgery features.

### 4.1. Gastric Surgery

Traditional surgical procedures, including the treatment of UGI cancers, are increasingly finding endoscopic alternatives. Specifically, ESD for the removal of early gastric cancers has been shown to be safer and more effective compared to conventional surgery, when performed by expert operators [61]. Endoscopic sleeve gastroplasty showed significantly less adverse events and good clinical outcomes [62], although it reported a less weight loss compared to the conventional laparoscopic sleeve gastrectomy [63]. Patients had significantly lower rates of adverse events compared with the laparoscopic sleeve gastrectomy ones (5.2% vs. 16.9%); the body weight loss ratio (compared with baseline) was also lower in the endoscopic sleeve gastroplasty group compared with the laparoscopic sleeve gastrectomy group (17.1 ± 6.5% vs. 23.6 ± 7.6%). Per-Oral Endoscopic Myotomy (POEM), as a treatment, has been suggested to be superior to Heller myotomy in short term efficacy for treating achalasia [64]. Although these are mature technologies, international experience is variable, and services are slowly developing to offer these as alternatives procedures for patients [65]. Given the time-consuming nature and skills required for these advanced endoscopic procedures, they are an appealing target for robotic assistance.

### 4.2. Novel Flexible Surgical Robots

Robotics for UGI applications is predominantly centred around access, stability, and instrumentation. Various methods have been attempted and are in ongoing development. For the UGI surgery, devices predominantly have two arms mounted on the tip of a flexible endoscope. These are operated independently and often require a second operator to control and act as the surgeons two hands for the procedure [66].

**Figure 3:**
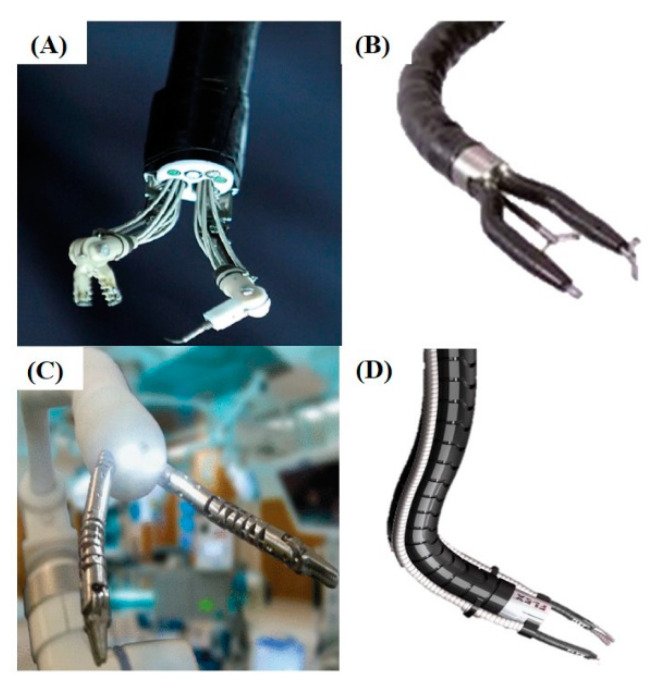
Novel flexible robots: (**A**) EndoMaster’s robotic system (EndoMaster Pte, Singapore) [67]; (**B**) ENDOSAMURAI^TM^ Olympus Medical Systems Corp. (Tokyo, Japan) [69]; (**C**) i^2^Snake robot [70,71]; (**D**) Flex^®^ Robotic System (Medrobotics Corp., Raynham, MA, USA; courtesy of Medrobotics Corp.) [72].

The EndoMaster’s robotic system (EndoMaster Pte, Ltd., Singapore) is based on a master device remotely controlling the surgical “slave” devices, through an endoscope (Figure 3A). This has been used for human gastric ESD, operated remotely, with favourable outcomes in 2012 [67]. The mean submucosal dissection time was 18.6 min (median of 16 min, range between 3–50 min). No perioperative complications were encountered. However, it has not yet been granted FDA or CE marks approval because of the questions in sterilization procedures [68]. Potential virus or bacterial residues can cause cross-infection, especially when infectious diseases, e.g., COVID-19, are prone to pandemics.

The ENDOSAMURAI™ from Olympus Medical Systems Corp. (Tokyo, Japan) has arms mounted on the end of the endoscope (Figure 3B). An overtube can then be used to stabilize the system allowing for more precise actions [69]. This device has been used to perform a number of procedures in animal studies including intra-abdominal exploration and transgastric small bowel resection [73]. In in-vivo conditions, the total time of the procedure, from the beginning of the resection until the completion of the anastomosis in small bowel wall, was on average 110 min (range: 90–125 min) and leak pressure was 53 mmHg.

The direct drive endoscopic system (Boston Scientific Corp., Natick, MA, USA) uses arms mounted on an overtube device, requiring two operators: one to perform a conventional endoscopy and one to manage the arms. This has been used in anaesthetised pigs for endoscopic mucosal resection [74]. It took at least 1 h and 26 min to perform the endoscopic mucosal resection. No complications of bleeding, perforation or hemodynamic compromise occurred. Inspection of the excised stomach showed no full thickness damage or serosal changes.

The i^2^Snake robotic platform has 4 channels within a robotic arm, which allows for 2 robotic instruments, a camera and a light source [70,71]. This system was designed for Ear–Nose–Throat procedures but has potential for more complicated procedures, such as ESD or POEM (Figure 3C). The robot showed high repeatability in executing the task with a standard deviation of 1.3 mm around the mean retroflexion trajectory, and a maximum displacement of 2.5 mm. The flexible robot is able to reach a complete retroflexion within a diameter of 110 mm.

The Flex^®^ Robotic System (Medrobotics Corp., Raynham, MA, USA) (Figure 3D) uses a transoral approach for operations of the oropharynx and primarily for the Ear–Nose–Throat procedure. This endoluminal robot is an operator controlled flexible endoscope system that includes a rigid endoscope and computer-assisted controllers, with two external channels for the use of compatible 3.5 mm flexible instruments [72]. Epiglottectomies were successfully performed and the average time of the procedure was 42 min (number of trials is 5, standard deviation is 28 min) [75].

Robotic assistance with flexible endoscopes for increasingly complex UGI surgery is a rapidly expanding field and there are several competing lines. There are hurdles to overcome in finding the optimal system, which is driving the ongoing evolution of these robotic devices.

## 5. Artificial Intelligence in Gastroscopy

Over 10% of cancers within the UGI tract are missed on endoscopy [12]. This is clearly a concern for endoscopists and patients, making computer-aided diagnostics (CAD) an appealing mechanism to minimize these missed lesions.

“Hand crafted” algorithms allow characteristic features of a lesion to be programmed so that these can be “recognized” by the system. Deep learning algorithms for image recognition, by comparison, begin to allow an automated extraction of these patterns [76]. Increasingly complex convolutional neural networks (CNNs) allow grouping of images by similarities, leading to classification and “recognition”. The main targets within the UGI tract so far have been Barrett’s oesophagus, oesophageal and gastric cancer and helicobacter infection [76,77].

Using this technique for the image recognition of gastric cancer results in a superior diagnostic accuracy compared to non-expert endoscopists, and similar to the expert ones [78]. At present, systems are sensitive to abnormality but have poor specificity of picking out relevant mucosal change [79]. Using deep learning, diagnostic sensitivity and specificity improve with the increase of balanced data. Although it would be less likely to perform better than a highly skilled endoscopist, if he/she is not fatigued and if he/she is in a non-distracting environment, AI may represent an important diagnostic aiding tool. Indeed, AI can guarantee a high reproducibility and scalability, paying the way for a standardization of diagnostic outcomes with a possible reduction of costs due to the extensive and required training programs.

In Barrett’s oesophagus, images containing neoplasia were recognized with more specificity and sensitivity by a CAD system than a human endoscopist [80]. For oesophageal squamous cell carcinoma deep learning through CNNs results in rapid image analysis with 98% sensitivity. Again, the specificity was relatively poor. The CNN was also able to distinguish between superficial and advanced disease with an accuracy of 98% [81]. Such systems are aimed at improving detection of early neoplasia for a real-time clinical use [82,83]. Given the high miss rate for precancerous lesions and oesophageal squamous cell carcinoma, the introduction of CAD is highly desirable. In helicobacter detection, machine learning algorithms have been seen to be more sensitive and specific than a human endoscopist, with a sensitivity of 86.7% and a specificity of 86.7%, compared to 75% and 63% demonstrated in studies using conventional endoscopy [84,85]. Moreover, a CNN-based computer-aided detection has been recently developed for a better assessment of tissue invasion depth in early gastric cancer achieving 89.16% of accuracy and 76.47% of specificity [86]. WCE often requires lengthy manual review of many thousands of images; CNNs have been suggested to have 100% sensitivity and specificity in recognizing coeliac disease from controls [87]. Their ability to rapidly analyses images, without fatigue makes this an ideal platform. As such, CAD could be effectively utilized to reduce the workload in capsule endoscopy. Potentially, if coupled with other technologies, such as a magnetic capsule endoscope, AI could be used to create a minimally invasive diagnostic test for the UGI tract and minimize the need for the physician’s input.

Primarily, CAD is used as an aid to the endoscopist in the hope that fewer lesions will be missed, especially by non-expert endoscopists. Differences have allowed a faster development and clinical application of AI in the lower GI tract [76]. The higher incidence of cancers and clear-cut dysplasia isolated on lower GI surveillance allowed a large databank to be gathered for learning quickly. The nature of dysplasia within the lower GI tract is often more easily distinguished, with a well demarcated polyp formation. This compared to the UGI tract, in which dysplasia is often formed on the background of an inflammation or on abnormal tissue, which is more difficult to distinguish [88,89,90,91]. Although it has taken longer to build AI and CAD for the UGI tract, this is also following rapidly and will benefit of what is already performed today for the lower GI tract.

The potential for this automated machine learning in AI seems limitless, but there are difficulties in real world implementation, which could soon be overcome with the new collaboratively conducted prospective and multicentre studies, obtaining large scale and high-quality image data [92]. Examples of such trials can be found in the “clinicaltrials.gov—National Institute of Health (NIH)” registry, including very recent, cohort, prospective, randomized multicentre studies of AI-assisted digestive endoscopy, aimed at recruiting 3600 participants in China [93].

As such AI could truly improve the quality of UGI investigation and the detection of UGI lesions as it has already shown to outperform humans in endoscopic image recognition.

## 6. Endoscopy Services in the COVID-19 Era: Call for Innovation

The impact of suspended elective endoscopy procedures at this time of the current COVID-19 pandemic could significantly affect gastrointestinal health [94,95]. With this in mind, the new consensus guidance using the modified Delphi process aimed to facilitate a rapid and safe reopening of global endoscopy services has been recently developed [94].

However, the threat of healthcare-associated infections and the recent lockdown of endoscopy provision due to SARS-CoV-2 has prompted a careful look into other ways of dealing with endoscopy requests [96]. UGI endoscopy remains an aerosol-generating procedure, due to the potential for retching, coughing and open airway suction use. It is therefore one of the main areas where new processes and protocol are needed [97]. In the midst of the pandemic, forgotten or new pathways and protocols have been brushed up and discussions about employing new, distance-retaining, non-aerosol generating procedures are much alive.

Apart from recent universally accepted consensus statements to guide the recovery of endoscopy units in the COVID-19 era, there is certainly a renewed interest in robotic and telemedicine provisions in gastroenterology. However, even now telemedicine seems still limited to follow-up care of patients with inflammatory bowel and liver disease [98,99]. In the light of recent developments in the field of mobile-cloud-assisted, wireless body sensors and remote robotic endoscopic technologies, patients could undergo a spectrum of various diagnostic procedures amid their daily routines [100,101,102]. Internet-based remote visit has been proofed as an effective and safe option to deliver care at a distance, limiting the risks of infection [103]; a detailed description of wireless body sensors is out of the scope of this review and has been extensively reviewed in [104,105].

**Figure 4:**
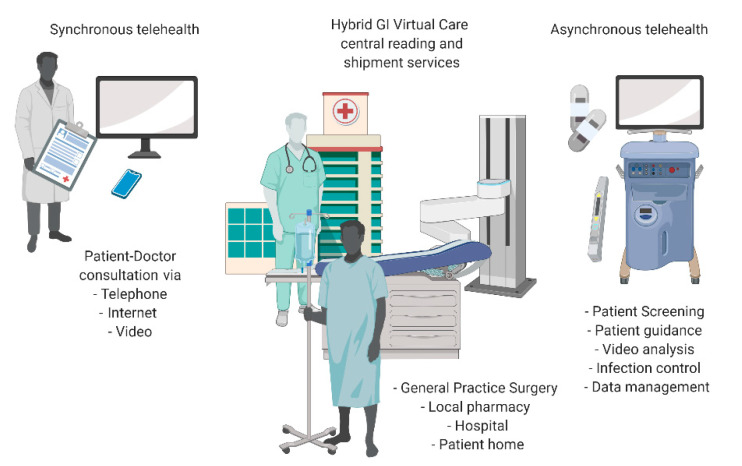
Remote health consultation (created with BioRender [110]).

The web-based, patient–doctor communication tools enable data and video transfer and analysis centralizing the resources of the medical experts with a consequential significant reduction of capital expenditure [106]. This model enables adoption of future innovations, such as Internet of Things (IoT) technologies, smart healthcare, robotics, AI-based video analysis and remote consultation (Figure 4). The development of different IoT sensors may enhance the current endoscopy techniques and allow for remote and real-time functioning of modern endoscopy centres [107]. Together with smart health-care concept, it allows for robotic equipment to provide remote monitoring and diagnosis of patients in real-time [104]. Future 5G communication networks and smart miniaturized antennas will facilitate the widespread use of these concepts. Robotic gastroscopes and other next-generation endoscopic devices could supplement medical diagnosis in various locations, including nursing and patients’ own homes [108,109].

Probably, the current pandemic will affect how medical healthcare functions for a long time, which calls for innovation and thinking outside the box. In the era of a growing number of patients with oncologic and other non-communicable and communicable diseases, the practice of gastroenterology needs novel solutions. As minimal-contact robotic platforms are already available, their further development and implementation into running endoscopy programs are essential.

## 7. Conclusions and Discussions

UGI tract disorders are among the most common diseases worldwide. Gastric and oesophageal cancers are both internationally common but also carry a poor prognosis when compared to many other malignancies. Therefore, it is of great benefit to patients that biological tissue abnormalities can be diagnosed and treated in the early stage. The diagnosis and treatment schemes using gastroscopes, as the main tool in UGI tract, have been continuously developed because of its easy access to the human body.

Up to now, there are various devices, both commercially available and at mature research levels, for treatment of the UGI tract. Commercial gastroscopes are mostly conventional manually actuated tethered gastroscopes and capsule gastroscopes. So far, manually actuated tethered gastroscopes are generally driven by cables, with a relatively small diameter and a hand-held control. However, it is common to cause discomfort to the patient while inserting them into the digestive tract. The commercially available robotic gastroscopes—potentially inspired by research examples—are mainly magnetically actuated capsule gastroscopes, which can easily negotiate the gastric cavity without discomfort or sedation for patients. In addition to commercially available gastroscopes, researchers have been proposing innovative devices with alternative propulsion and bending mechanisms. The gastroscopes with soft-tether are mainly driven by the HydroJet-based, cables, and pneumatic mechanisms, whereas the wireless gastroscopes consist of self-driven capsule gastroscopes and magnetically actuated ones. The research-oriented gastroscopes explore more possibilities and future applications. In addition to describing the technical features of robotic gastroscopes, this review article also details the outcomes of these devices in clinical practices. Both the technical features and clinical outcomes of robotic gastroscopes support clinicians and researchers to have further room for application and development. In addition to diagnosis, the gastroscope also expands into the field of treatment and surgery. This article also discusses some of the robotic surgical devices used for UGI complex interventions. The performance of various surgical instruments was compared, and the current status quo was deeply considered.

We are now in an era full of changes, and these have exceeded the expectations of most of us. The most relevant fact, at present, is that new sources of infection, e.g., SARS-CoV-2, are interrupting our normal living conditions, and also medical conditions. Embracing these changes with a positive attitude could lead to further development in the future, giving us a greater possibility to improve the quality of life for society. The use of the robotic gastroscope has greatly enhanced the diagnosis and surgery of the UGI tract, allowing higher accuracy and visualization capabilities. However, more in-depth research based on the current status quo will definitely improve the performance. Digitalization, telemedicine and AI are intense development trends in recent years. The combination of AI and robotic gastroscopes will undoubtedly make diagnosis and treatment more intelligent. The large-scale application of robotics and AI can effectively solve the “social and medical distance” problem at this stage. It can reduce the frequency of contact or even realize “zero” contact on the premise of ensuring the diagnosis and surgical effect. Furthermore, in low- and middle-income countries, the significance is far beyond that. Due to the long-term lack of medical resources (especially expert doctors), diagnostic and therapeutic services only cover a very limited population. The promotion of AI-assisted and teleoperated robotic gastroscopes will allow more people to have access to an efficient, standardized, and reliable diagnosis and surgery.

## Abbreviations

AI	Artificial Intelligence
CAD	Computer-Aided Diagnostics
CNNs	Convolutional Neural Networks
COVID-19	COronaVIrus Disease 19
ESD	Endoscopic Submucosal Dissection
FPS	Frame Per Second
IoT	Internet of Things
LED	Light Emitting Diode
NOTES	Natural Orifice Transluminal Endoscopic Surgery
POEM	Per-Oral Endoscopic Myotomy
SARS-CoV-2	Severe Acute Respiratory Syndrome-CoronaVirus-2
UGI	Upper GastroIntestinal
WCE	Wireless Capsule Endoscope
